# High prevalence of protein tyrosine phosphatase non-receptor N22 gene functional variant R620W in systemic lupus erythematosus patients from Kuwait: implications for disease susceptibility

**DOI:** 10.1186/s41927-018-0015-x

**Published:** 2018-03-16

**Authors:** Adel M. Al-Awadhi, Mohammad Z. Haider, Jalaja Sukumaran, Sowmya Balakrishnan

**Affiliations:** 1 Department of Medicine, Faculty of Medicine, Kuwait University, Jabriya, Kuwait; 2 Rheumatic Disease Unit, Al-Amiri Hospital, Dasman, Kuwait; 3 Department of Pediatrics, Faculty of Medicine, Kuwait University, P. O. Box 24923, 13110 Safat, Kuwait

**Keywords:** Protein tyrosine phosphatase non receptor-22, Systemic lupus erythematosus, Kuwait, Gene, Functional variant

## Abstract

**Background:**

Systemic lupus erythematosus (SLE) is an autoimmune inflammatory disease which involves the loss of self-tolerance with hyperactivation of autoreactive T- and B-cells. Protein tyrosine phosphatase non-receptor type 22 (PTPN22) encodes for lymphoid specific phosphatase (LYP) which is a key negative regulator of T lymphocyte activation. The aim of this study was to investigate the association between *PTPN22* gene functional variant R620W and systemic lupus erythematosus (SLE) by comparing its prevalence in Kuwaiti SLE patients and controls.

**Methods:**

The study included 134 SLE patients and 214 controls from Kuwait. The genotypes of *PTPN22* gene functional variant R620W were determined by PCR-RFLP and confirmed by DNA sequence analysis in both SLE patients and the controls.

**Results:**

A relatively high prevalence of the variant 620 W (T-allele) of the *PTPN22* gene was detected in the SLE patients from Kuwait. 35.7% of the SLE patients had at least one variant allele (T-allele) compared to 15.9% in the controls. A statistically significant difference was detected in the frequency of variant genotypes, TT and CT between SLE patients and the controls (*p* < 0.0001). No association was detected between the *PTPN22* gene variant and the Raynaud’s phenomenon, renal involvement and severity of the SLE.

**Conclusions:**

The frequency of *PTPN22* gene functional variant R620W reported in this study is amongst the highest compared to other world populations. A high prevalence of this variant in SLE patients in comparison to the healthy controls suggests its significant contribution in conferring susceptibility to SLE together with other factors.

## Background

Systemic lupus erythematosus (SLE; MIM 152700) is a common, complex autoimmune disease with multiple-organ involvement, characterized by the production of pathogenic autoantibodies directed against cytoplasmic and nuclear cellular components [1]. It occurs predominantly in women (> 90%) and is distinguished by a loss of tolerance to self-antigens, the deposition of immune complexes and tissue inflammation and destruction. There are variable clinical manifestations associated with SLE which include arthralgia, rashes, alopecia, serositis, leukopenia and renal involvement [2, 3]. Unrestricted hyper-activation of the immune system may lead to the overproduction of autoantibodies, immune complex deposition, inflammatory cytokine release and eventually organ damage in SLE pathogenesis [4, 5]. The causes of SLE are not completely understood at present. It is thought that the onset of SLE results from multifactorial etiology, involving hormonal factors, environmental triggers and genetic susceptibility [6]. Systemic autoimmune diseases e.g. SLE and many others are thought to share common genetic causative factors in populations of different ethnic or racial origin [7]. The involvement of major histocompatibility complex (MHC) also supported this hypothesis. However, non-MHC alleles such as a functional polymorphism (rs2476601, Arginine 620 Tryptophan, R620W) in protein tyrosine phosphatase non-receptor type 22 (PTPN22), which encodes the lymphoid PTP (LYP), was reported to be significantly associated with multiple autoimmune disorders including type 1 diabetes mellitus (T1DM; [8]), rheumatoid arthritis [9], SLE [10], Hashimoto thyroiditis [11], autoimmune thyroid disease [12] and Grave’s disease [13].

The *PTPN22* gene encodes a lymphoid-specific phosphatase (LYP) which has been shown to be a negative regulator of T cell activation [14]. The LYP contribute to T cell activation by binding to the regulatory Src tyrosine kinase (Csk) [14]. It has been reported that a functional variant R620W (C1858T polymorphism) of the *PTPN22* gene is located in a motif which facilitate binding of the LYP with Csk [15]. The variant form, 620 W carries a tryptophan residue in place of arginine (R) and affects the binding of LYP and Csk thereby disrupting the regulation of T cell receptor-signaling kinases e.g. Lck, Fyn and ZAP70 [14–16]. It has been postulated that the individuals carrying the variant allele (620 W) have an altered threshold for thymic selection, with increased numbers of autoreactive T cells escaping the negative selection, thus persisting in the circulation, and are prone to autoimmunity [17]. The C1858T variant has also been shown to be associated with changes in cytokine profile in SLE patients in vivo [18].

In spite of a large number of reports linking the *PTPN22* gene R620W functional variant with a number of autoimmune diseases, conflicting results have appeared in the literature about its prevalence in different world populations [3, 5, 7, 19–22]. In view of these diverse findings on prevalence and relationship of the *PTPN22* gene R620W functional variant with SLE, we carried out this study in SLE patients from Kuwait to investigate its possible association with susceptibility to SLE in a completely different population/ethnic group.

## Methods

The SLE patients were recruited from two major teaching hospitals (Amiri and Mubarak Al-Kabeer) from Kuwait. The inclusion criteria described by the American College of Rheumatology (ACR) for the diagnosis of SLE was used [23]. For the patients included in the study, a diagnosis of SLE had been made at least 2–3 months prior to their recruitment. The information collected from the patients included age, age-at-onset, gender, clinical manifestations associated with SLE and disease severity. The evaluation of SLE severity was made as follows: the patients were considered to have a ‘mild disease’ if they presented muco-cutaneous serositis, and/or arthritis and ‘mild-severe’ disease if the patients had hematological, renal and neurological manifestations. The renal disease associated with SLE was ascertained if the 24 h urine protein excretion exceeded 500 mg or hematouria was detected (> 5 red blood cells/field). The hematological abnormalities associated with SLE included the presence of hemolytic anemia with reticulocytosis, or leucopenia (< 4000/mm^3^ on > two occasions), or lymphopenia (< 1500 mm^3^ on > two occasions) or thrombocytopenia (< 100,000/mm^3^ in the absence of drugs). The presence of seizures or psychosis without the use of drugs or metabolic derangement were considered as the neurological presentations associated with SLE. The frequency of *PTPN22* gene polymorphism in SLE patients was compared to that in a group of 214 healthy controls, matched for age and sex with the patients. The controls subjects were unrelated to the patients; were otherwise healthy and were seen at hospital outpatient clinics for minor illnesses. The controls were thoroughly examined by a specialist to ascertain their health status before recruitment in the study.

### Identification of genotypes for *PTPN22* gene functional variant (R620W)

The *PTPN22* gene polymorphism genotypes were determined in 126 patients with SLE and 214 healthy controls. Approximately 5 ml blood was collected from all the study subjects in appropriate tubes. For molecular studies, blood was anti-coagulated in the presence of EDTA. Total genomic DNA was isolated by using a standard method [24]. The genotypes for a non-synonymous single nucleotide polymorphism (SNP) +1858C➔T (rs2476601) in the *PTPN22* gene were identified by polymerase chain reaction-restriction enzyme fragment length polymorphism (PCR-RFLP) method as described earlier [25]. A 218 bp DNA fragment was amplified by using the primers: Forward primer: 5’-ACTGATAATGTTGCTTCAACGG-3′ and reverse primer: 5-TCACCAGCTTCCTCAACCAC-3′. The PCR mixture contained 10× PCR buffer (Applied BioSystems, Foster City, USA); 1.5 mM MgCl_2_; 0.2 mM of each of the dNTPs (deoxyribonucleotide triphosphates); 20 pmol of each primer, 250 ng template DNA and 1 U AmpliTaq DNA polymerase (Applied BioSystems). The amplification conditions used were denaturation at 94 °C for 5 min followed by 35 cycles of 94 °C for 30 s, 60 °C for 30 s and 72 °C for 30 s and an extension step at 72 °C for 5 min. The PCR product was digested with restriction enzyme *Rsa*I at 37 °C for 90 min. The cleavage products were analyzed by 2% agarose gel electrophoresis and visualized under UV light after staining with ethidium bromide. The 218 bp PCR product did not have an *Rsa*I cleavage site when 1858 T allele (620 W variant) was present and the presence of C1858 allele (R620) was associated with the presence of 176 bp and 42 bp cleavage products. In case of a heterozygous individual both the 218 and 176 bp bands were detected. The PCR amplicons were sequenced in a blinded manner on ABI 3130 genetic analyzer to confirm the genotypes. Genotypes could not be done in 8 SLE patients due to low volume of the blood samples obtained. The study was carried out according to the Helsinki declaration and was approved by the Institutional Ethics Committee for the protection of human subjects in research. Written informed consent was obtained from all the study subjects.

### Statistical analysis

The information and the data collected from and on the study subjects was analyzed using the Statistical Package for the Social Sciences version 24 (SPSS, Chicago IL, USA). The frequencies of various genotypes and alleles detected among the SLE patients and controls were calculated by direct counting. The significance of their association was evaluated by using the Chi-square test, Fisher’s Exact test and *p*-values were regarded significant when < 0.05. The strength of association was estimated by the odds ratios which were calculated at 95% confidence interval. The genotype distribution was also tested for Hardy Weinberg equilibrium by goodness of fit method using MSTAT software.

## Results

This study included 134 SLE patients and 214 controls. In the patients group (*n* = 134), there were 123 (91.8%) females and 11 (8.2%) males. In the control group, there were 199 females. In the SLE patients group, the age of onset information was available in 118 cases. When stratified on the basis of age of onset, 16/118 (14%) patients were aged between 1 and 14 years, 25/118 (21%) between 15 and 24 years, 26/118 (22%) between 25 and 34 years, 34/118 (29%) between 35 and 44 years and 17/118 (14%) > 45 years respectively. The data on SLE patient characteristics and clinical manifestations has been presented in Table 1. The genotype and allele frequencies of *PTPN22* gene R620W functional variant amongst the SLE patients and controls have been presented in Table 2. A comparison of genotype frequency between SLE patients and the controls showed statistically significant differences in the case of CC and TT genotypes (Table 2). Similarly, the frequencies of C- and T-alleles were also significantly different between the SLE patients and controls (Table 2). Statistically significant differences were detected in all the three genotypes (CC, CT, TT) between females and male SLE patients (*P* < 0.0001 in case of all three genotypes). However, as it is generally the case, the incidence of SLE was much higher in females than in the males in our study group. A comparison was also made for the genotype distribution on the basis of gender between the SLE patients and the controls (Table 3). For the CC and CT genotypes, the difference was statistically significant both for the females and males between the SLE patients and that in the controls. However, in the case of TT genotype, there was no significant difference between the genotype distribution between SLE patients and the controls in relation to their gender (Table 3). It may be mentioned here that in the case of controls, only 2/214 subjects had a TT genotype and of these, one was male and the second a female. We did not find an association between T-allele of the *PTPN22* gene functional variant and the Raynaud’s phenomenon, nephritis or the disease severity in SLE or any of its other manifestations in patients from Kuwait (data not shown).

**Table 1:** The characteristics of systemic lupus erythematosus (SLE) patients included in the study (*n* = 134). S.D., standard deviation; ANA, anti-nuclear antibody

Gender ratio (Female: Male)	11: 1 (123/11)
Mean age (± S.D.), years	36.7 (± 9.3)
Mean age at diagnosis (± S.D.), years	30.6 (± 8.3)
Mean disease duration (range), months	48 (6–280)
Clinical manifestations: n (%)	
Malar rash	73 (54.4)
Mouth ulcers	26 (19.4)
Photosensitivity	25 (18.7)
Discoid rash	9 (6.7)
Raynaud’s phenomenon	22 (16.4)
Arthritis	128 (95.5)
Serositis	29 (21.6)
Renal involvement	22 (16.4)
Hematological abnormalities	51 (38)
Neurological disorders	5 (3.7)
Immunological abnormalities^a^	95 (70.9)
ANA^b^	134 (100)
Hypertension^c^	7 (5.2)

^a^As per criteria of the American College of Rheumatology (ACR) [23]

^b^A standard indirect immunofluorescence method was used for detection and ascertainment as per ACR criteria and cutoff limits [23]

^c^Blood Pressure > 140/90 mmHg (or > 130/80 mmHg in the case of renal insufficiency)

**Table 2:** Genotype and allele frequency of *PTPN22* gene R620W functional variant in SLE patients and controls

Genotype/Allele	SLE patients *N* = 126 (%)	Controls *N* = 214 (%)	OR (95% CI)^a^	*P*-value*
CC	81 (64.3)	180 (84.1)	0.34 (0.20–0.57)	< 0.0001
CT	23 (18.3)	32 (15)	1.27 (0.70–2.28)	0.51
TT	22 (17.4)	2 (0.9)	22.42 (5.17–97.21)	< 0.0001
Distribution of Alleles				
C – allele	185/252 (73.4)	392/428 (91.6)	0.25 (0.16–0.39)	< 0.0001
T – allele	67/252 (26.6)	36/428 (8.4)	3.94 (2.53–6.13)	< 0.0001

^a^OR, odds ratio at 95% confidence interval

*P-values were considered significant when < 0.05

**Table 3:** Comparison of genotypes of *PTPN22* gene R620W functional variant between SLE patients group and controls stratified according to gender

Genotype/Gender	SLE patients *N* (%)	Control *N* (%)	*P*-value^a^
CC	(*N* = 76)	(*N* = 177)	< 0.001*
Female	70 (92.1)	115 (65)	
Male	6 (7.9)	62 (35)	
CT	(*N* = 21)	(*N* = 32)	< 0.001*
Female	21 (100)	19 (59.4)	
Male	0 (0)	13 (40.6)	
TT	(*N* = 21)	(*N* = 2)	0.25
Female	19 (90.5)	1 (50)	
Male	2 (9.5)	1 (50)	

^a^Chi-square test of the full cohort

*P-value were considered significant when < 0.05

## Discussion

The most significant finding in this study is that a relatively high frequency of *PTPN22* gene functional variant 620 W was detected in the SLE patients (17.4%) compared to 0.9% in the controls. When taken together, the homozygous and heterozygous combinations of this variant allele were detected in 35.7% of the SLE patients from Kuwait. This is significantly higher than previous reports from other populations/ethnic groups. Machado-Contreras et al. [5], did not find homozygous TT genotype in any of their Mexican SLE patients, however heterozygous CT genotype was detected in 6% Mexican SLE patients. Similarly, in a Greek study on the SLE patients from the Island of Crete, TT genotype was not detected at all while CT genotype was detected in 5.39% cases. In a report on SLE patients from Egypt [26], the authors could not detect a homozygous TT genotype but did find heterozygous CT genotype in 52.5% SLE cases. A comprehensive *PTPN22* gene association study [3], reported minor allele frequency (MAF) of 11.9% in European Americans, 2.1% in African Americans and 7.6% in Hispanics. This study also reported that the association of the 1858 T allele with SLE was greater in European American patients with familial SLE (11.9%) compared to the sporadic SLE (8.2%). A study from Poland [21] found a positive association between the variant allele of the *PTPN22* gene and SLE. Aksoy et al. [22] did not find the TT genotype in Turkish SLE patients while the CT genotype was detected in 7% cases. Another study, on white Americans [27], reported that the TT genotype was detected in 2.5% SLE patients while the CT genotype was found in 20.4% SLE patients. In this study, the overall risk-allele frequency was found to be 12.67% compared to 8.64% in the white American controls. Positive association between *PTPN22* gene polymorphism and SLE susceptibility has been reported from Sweden [28] and Spain [29]. Namjou et al. [29] reported findings from genotyping of ten SNPs in four large multi-ethnic populations and using their results in conjunction with data from the Hap-Map project, concluded that SLE association with *PTPN22* was largely accounted for by the R620W variant (rs2476601) in individuals of European ancestry [21–26, 30, 31]. A strong North-South gradient in the risk-allele frequency has also been reported in Europe [32].

Recent reports provide information on the mechanism of R620W functional variant of the *PTPN22* gene [33–38]. In the light of postulated scheme for involvement of R620W variant in the molecular mechanism of autoimmunity [33, 34], our data from SLE patients from Kuwait supports and highlights its role as a significant determinant of the SLE susceptibility. However, it may also be appropriate to mention that the genetic factors which contribute to susceptibility/protection to develop SLE most likely involve multiple genes. Kuwait is a small country located in the North of Arabian Gulf. The population of Kuwait is quite diverse; Kuwaiti Arabs constitute nearly 45% of the population. There is a high incidence of consanguinity (54%; [39]), which often results in familial clustering of common chronic disorders. The incidence of diseases with autoimmune etiology such as type 1 diabetes mellitus is amongst the highest in the region and bordering with the high-incidence countries of the world [40, 41]. The original settlers of Kuwait were immigrants from Najd, an area that now constitute eastern and central Saudi Arabia. The ethnic origin of Kuwaiti Arabs is quite varied; 50% are of Arab origin, some are Bedouins and nearly 50% are immigrants [42]. The Arabs residing in most of the Gulf countries are a result of an admixture with other populations such as Persians, Turks, South Asians, Europeans and Africans [42]. The genotype frequency of variant 620 W reported in our SLE patient group from Kuwait is amongst the highest (TT, 17.4% and combined TT and CT in 35.7% patients) compared to any of the other populations/ethnic groups. This can possibly be due to a cumulative effect of unique genetic/ethnic background along with a very high rate of consanguinity (54%) in the Kuwaiti population and can at least in part, explain the high incidence of autoimmune diseases including SLE in the country.

## Conclusions

The frequency of *PTPN22* gene functional variant R620W reported in this study is amongst the highest compared to other world populations. A high prevalence of this variant in SLE patients in comparison to controls suggests its significant contribution in conferring susceptibility to SLE along with other factors.

## Abbreviations

Csk: Src tyrosine kinase
DNA: Deoxyribonucleic acid
dNTP: Deoxyribonucleotide triphosphate
LYP: Lymphoid specific phosphatase
MHC: Major histocompatibility complex
PCR: Polymerase chain reaction
PTPN22: Protein tyrosine phosphatase non-receptor type 22
R: Arginine
RA: Rheumatoid arthritis
RFLP: Restriction fragment length polymorphism
SLE: Systemic lupus erythematosus
SNP: Single nucleotide polymorphism
T1DM: Type 1 diabetes mellitus
W: Tryptophan
